# Array antenna with series-fed configuration providing high radiation performances for automotive radar in IoT applications

**DOI:** 10.1038/s41598-026-40981-x

**Published:** 2026-04-01

**Authors:** Hassan Zakeri, Mahdi Parvaneh, Gholamreza Moradi, Mohammad Alibakhshikenari, Bal S. Virdee, Mariana Dalarsson

**Affiliations:** 1 Department of Electrical Engineering, Amirkabir University of Technology, 424 Hafez Ave, Tehran, 15875-4413 Iran; 2 LERO, The Research Ireland Centre for Software, College of Science and Engineering, School of Computer Science, University of Galway, H91 TK33 Galway, Ireland; 3 Department of Electrical and Electronics Engineering, Dogus University, 34775 Umraniye, Istanbul Türkiye; 4 Department of Electrical Engineering, Center for Communications Technology, London Metropolitan University, N7 8 DB London, UK; 5 School of Electrical Engineering and Computer Science, KTH Royal Institute of Technology, 100-44 Stockholm, Sweden

**Keywords:** Automotive radar sensors, High-gain antenna, PSADEA optimization, Internet of Things, Series-fed array, Engineering, Mathematics and computing

## Abstract

This paper presents two high-performance series-fed antenna array configurations optimized for 24 GHz automotive radar in Internet of Things applications. The $2\times 5$ and $4\times 5$ array designs employ circular microstrip patches, excited via a custom-designed power divider, to ensure uniform amplitude and phase distribution. A key contribution is the integration of the Parallel Surrogate-Assisted Differential Evolution Algorithm (PSADEA) to optimize antenna parameters, resulting in improved reflection coefficient, realized gain, and beamwidth. The proposed arrays achieve measured gains of 16 dBi and 19.5 dBi, with radiation efficiencies of 96.55% and 95.55%, respectively. Both arrays exhibit low return loss across the 23–25 GHz band and maintain a compact size suitable for integration into vehicle platforms. A comparative analysis with state-of-the-art designs confirms the effectiveness of PSADEA-based optimization in improving design performance with fewer electromagnetic simulations. Finally, the results demonstrate that the proposed arrays are strong candidates for automotive radar in IoT applications.

## Introduction

The rapid evolution of wireless communication systems has significantly expanded the scope of application for electronic technologies, particularly in the automotive sector. Among these developments, the push toward fully autonomous vehicles has emerged as a central focus. While the vision of fully self-driving cars is still under development, current advancements emphasize Advanced Driver Assistance Systems (ADAS), which enhance safety and driver comfort. These systems rely on multiple sensors and Internet of Things (IoT) devices to provide real-time situational awareness and decision-making support, with automotive radar playing a pivotal role in their functionality^1,2^.

**Fig. 1:**
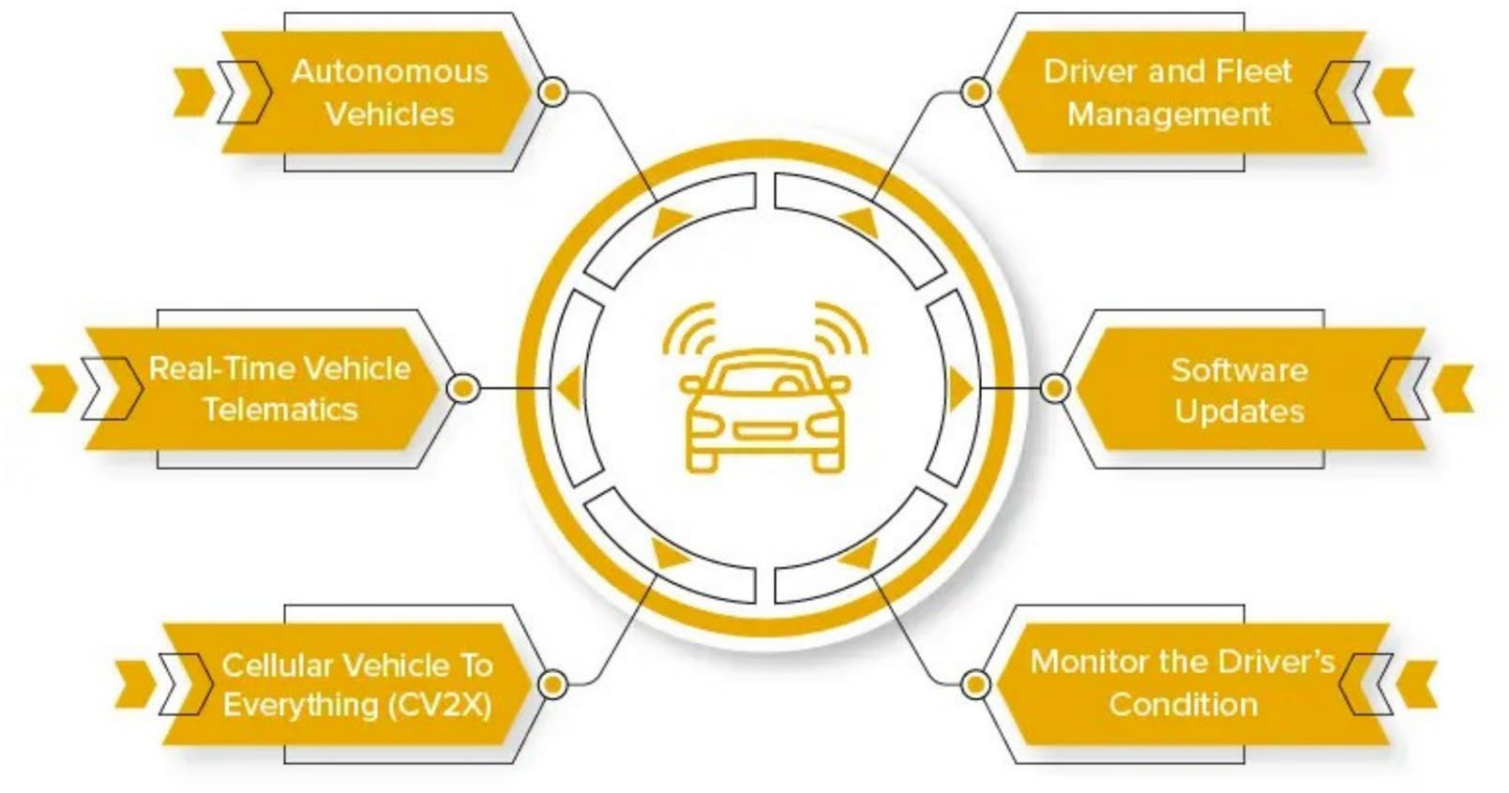
Use case benefits of real-time automotive radar in the IoT applications.

The increasing number of cars using different wireless devices to connect to the internet necessitates a high data transmission rate with minimal latency^3,4^. Due to this rise, current wireless communication technologies face increasingly complex design challenges^5^. Furthermore, as shown in Fig. 1, integrating automotive radar with IoT and artificial intelligence is set to transform various sectors, enabling reduced emissions, predictive maintenance, vehicle connectivity, innovative infrastructure, and fleet management.

Automotive radar technology has gained prominence across various industries due to its reliability and versatility. It is used not only in vehicle systems but also in applications such as vital sign monitoring, indoor localization, collision avoidance, and navigation. At the core of these radar systems are the antennas, which serve as the primary radiating components. Automotive radar antennas, particularly those based on planar and microstrip technologies, have proven effective due to their low profile, ease of integration, and directional radiation capabilities^6,7^.

In modern intelligent transportation systems, automotive radars are an integral part of the IoT ecosystem. Vehicles equipped with radar sensors act as distributed perception nodes, continuously collecting range, velocity, and occupancy information from their surroundings. This information can be shared with other vehicles, roadside units, or cloud-based management platforms via IoT communication links, enabling functions such as cooperative collision avoidance, traffic-flow monitoring, automated parking systems, and smart-infrastructure management. Consequently, the radar hardware used in vehicles not only serves traditional onboard safety tasks but also contributes sensing data to IoT applications, which explains the relevance of the use cases illustrated in Fig. 1.

With the increasing deployment of autonomous and semi-autonomous vehicles, there is a growing demand for radar systems capable of detecting objects over ranges of tens to hundreds of meters. The 24 GHz frequency band is widely used in automotive radar in IoT applications due to its favorable propagation characteristics and reliability under diverse weather and environmental conditions. Studies show that 24 GHz radar sensors and IoT devices can effectively detect objects at ranges of 30–100 m, making them well-suited for urban driving scenarios and short-to-mid-range applications^8–10^.

In addition to radar, ultrasonic sensors are employed for very close-range applications, typically under 10 m, making them ideal for parking assistance and low-speed maneuvering^11^. Meanwhile, narrowband sensors are often used for blind-spot detection (BSD) and short-range obstacle detection, reliably identifying objects at ranges of 30–50 m. These sensors are exceptionally efficient in cluttered environments where precision is crucial to minimize false detections^12^.

To meet these requirements, microstrip patch antennas are commonly adopted due to their lightweight construction, low cost, flexibility, and compatibility with printed circuit technologies. Among these, high-gain antenna arrays are preferred for automotive radar in IoT applications because they enable narrow beamwidths, thereby improving angular resolution and detection accuracy. This allows the system to distinguish between closely spaced targets, such as vehicles, pedestrians, or roadside objects^13,14^.

Various approaches to 24 GHz automotive radar antennas have been reported in the literature, aiming to meet stringent requirements for high gain, compact size, low loss, and ease of integration. Low-profile planar microstrip arrays have been widely investigated due to their compatibility with printed circuit board technology and vehicle-mounted platforms, typically achieving gains of 14–15 dBi while maintaining compact form factors^15,16^. More recent studies have explored large-scale planar arrays to enhance gain and beam shaping further. For example, high-element-count microstrip arrays employing series-parallel feeding and amplitude tapering techniques have demonstrated gains exceeding 20 dBi, albeit at the expense of increased aperture size, feed-network complexity, and fabrication sensitivity^17,18^.

Beyond planar structures, fully metallic and waveguide-based antennas have also been proposed to improve radiation efficiency and power handling at millimeter-wave frequencies. Frequency-scanning and gap-waveguide antennas operating at 24 GHz have reported gains above 20 dBi with radiation efficiencies exceeding 80%. Still, these solutions often involve multilayer metallic structures, precision machining, and limited compatibility with low-cost automotive manufacturing processes^8^. In parallel, system-level developments, such as compact 24 GHz radar frontends integrated with software-defined radios, highlight the growing demand for antennas that not only deliver high performance but also support modularity, scalability, and seamless integration into sensing platforms^19^.

Despite these advances, several challenges remain. Achieving a high gain-to-size ratio while maintaining wide impedance bandwidth, high aperture efficiency, and low feed-network loss remains difficult, particularly for compact planar arrays. Many existing designs rely on large numbers of radiating elements or complex feeding structures, which increase loss, cost, and design complexity. These limitations motivate the development of compact, low-loss antenna architectures with efficient aperture utilization, which form the focus of this work.

This paper presents two novel series-fed microstrip patch antenna arrays designed for 24-GHz automotive radar within IoT-enabled intelligent-transportation systems. The arrays-implemented in $2\times 5$ and $2\times 5$ element configurations follow a unified design methodology but differ in aperture size to meet application-specific performance requirements. A compact, single-sided power divider is employed to ensure uniform excitation and generate stable linear polarization, producing a fan-beam radiation pattern suitable for seamless integration into modern automotive radar and IoT sensing platforms.

To optimize the antenna design, an AI-driven approach is employed using the Parallel Surrogate-Assisted Differential Evolution Algorithm (PSADEA)^20–22^. PSADEA is part of the SADEA algorithm family^23,24^, and supports optimization tasks involving up to 40 parameters. Compared to standard global optimization techniques, such as Particle Swarm Optimization (PSO) and Genetic Algorithms (GA), PSADEA demonstrates significantly faster convergence-up to 30 times faster -while delivering superior performance in terms of gain, beamwidth, and return loss. The remainder of this paper is organized as follows: Section II describes the antenna array structure and the optimization methodology. Section III presents the simulation and measurement results, including radiation patterns and gain performance. Finally, Section IV concludes the paper by presenting key findings and outlining future research directions.

## Antenna array design and discussion

**Fig. 2:**
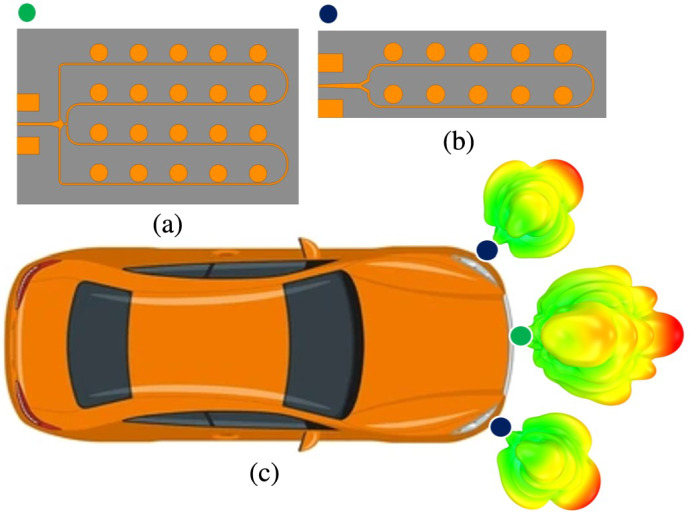
(**a**) Proposed $4\times 5$ series-fed antenna array, (**b**) proposed $2\times 5$ series-fed antenna array, and (**c**) illustration of antenna placement and corresponding radiation pattern for automotive radar in IoT applications.

The configuration of the proposed antenna, as demonstrated in Fig. 2, consists of two essential parts: the power divider and the radiating array patch elements.

The substrate layer used in the antenna is Rogers RT/Duroid®5880, with a dielectric constant of $\epsilon _r$ = 2.2, a loss tangent of tan$\delta$ = 0.0009, and the substrate thickness (*H*) is 31 mil. The CST Microwave Studio is employed to simulate antenna performance.

### Power divider

The power divider plays a critical role in ensuring uniform excitation across all radiating elements in the array. It delivers the input signal to each feedline with equal amplitude and phase. This balance is essential for achieving symmetrical radiation patterns and high array efficiency.

For the $4\times 5$ array, a four-way microstrip power divider is implemented. Its layout consists of sequential branching microstrip lines with carefully controlled widths and lengths to maintain impedance matching throughout the network. Tapered transitions gradually change the width of microstrip lines, minimizing reflections and ensuring a smooth transition of electromagnetic energy. The design includes:

- Main trunk lines with wider widths for low impedance,
- Branch lines with narrower widths for impedance transformation,
- Quarter-wavelength impedance transformers and tapered microstrip sections to maintain matching at junctions and minimize standing wave formation.

For the $2\times 5$ array, a simplified two-way power divider is used to drive two rows of elements. While structurally simpler, the same design principles apply, including the use of impedance transformers and tapering for efficient signal delivery. The performance of the power divider is critical: any amplitude or phase imbalance across the outputs would degrade the main lobe shape, increase side-lobes, and reduce gain.

**Fig. 3:**
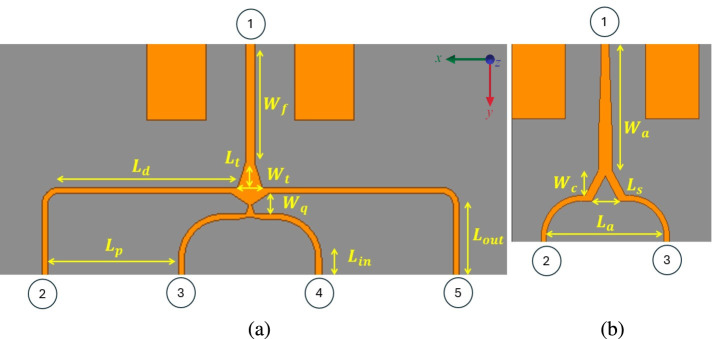
Schematic of the proposed power divider (**a**) $4\times 5$ ($W_{f}$= 8.2 mm, $W_{t}$= 1.7 mm, $L_{t}$= 1.6 mm, $W_{q}$= 1.4 mm, $L_{d}$= 12.1 mm, $L_{p}$= 8.85 mm, $L_{in}$= 3.9 mm, $L_{out}$= 7.5 mm) (**b**) $2\times 5$ ($W_{a}$= 9 mm, $W_{c}$= 1.8 mm, $L_{s}$= 2mm, $L_{a}$= 8.8 mm).

The $2 \times 5$ antenna array has overall dimensions of 63 mm $\times$ 19 mm, while the $4 \times 5$ antenna array measures 65.6 mm $\times$ 41 mm. The $2 \times 5$ array includes four parallel microstrip feed lines, and its feeding network is designed using a four-way power divider, as shown in Fig. 3a. For the $2 \times 5$ array, a simpler one-to-two power divider is employed with two feed lines, illustrated in Fig. 3b. To minimize power reflections and improve transmission efficiency, tapered transitions are implemented. These tapered lines enable smooth impedance transitions between different microstrip widths and characteristic impedances. Additionally, narrow transitional microstrip sections are inserted to reduce reflection coefficients further and improve impedance matching^25,26^.

To further quantify the feed network’s performance, the standalone two-way and four-way power divider structures were simulated separately to obtain their scattering parameters. Across the 23–25 GHz band, the simulated amplitude imbalance is below 0.35 dB, the phase imbalance remains within $4^\circ$, and the insertion loss is less than 0.5 dB, consistent with the low-loss characteristics of Rogers RT/Duroid®5880 at millimeter-wave frequencies. While direct probing of the integrated dividers was not feasible, the measured realized gain of both arrays agrees with simulation within 0.2 dB, confirming that the feed network introduces minimal loss and maintains proper amplitude and phase balance. Any significant imbalance or excess loss would noticeably degrade the main beam shape or reduce gain by several decibels, neither of which is observed in the measured results.

### Radiating elements

The radiating section of the antenna array, shown in Fig. 3, consists of circular microstrip patch elements arranged in either a $2\times 5$ or $4\times 5$ configuration. These patches are excited using proximity coupling, where each feedline runs beneath or beside the patch without direct electrical contact. This method offers several advantages:

- Improved bandwidth compared to edge-fed or probe-fed configurations,
- Reduced mechanical complexity during fabrication (no via or vertical transitions),
- Minimized undesired currents on the ground plane, improving polarization purity and pattern symmetry.

Each feedline is closely aligned with its corresponding patch, with a carefully controlled gap distance ($g_i$). These gaps are critical: they determine the coupling strength between the feedline and patch, which, in turn, affects the amount of radiated power, impedance matching, and bandwidth. A smaller gap increases coupling, potentially enhancing gain but risking degradation of return loss. Conversely, a larger gap may lead to poor excitation.

**Fig. 4:**
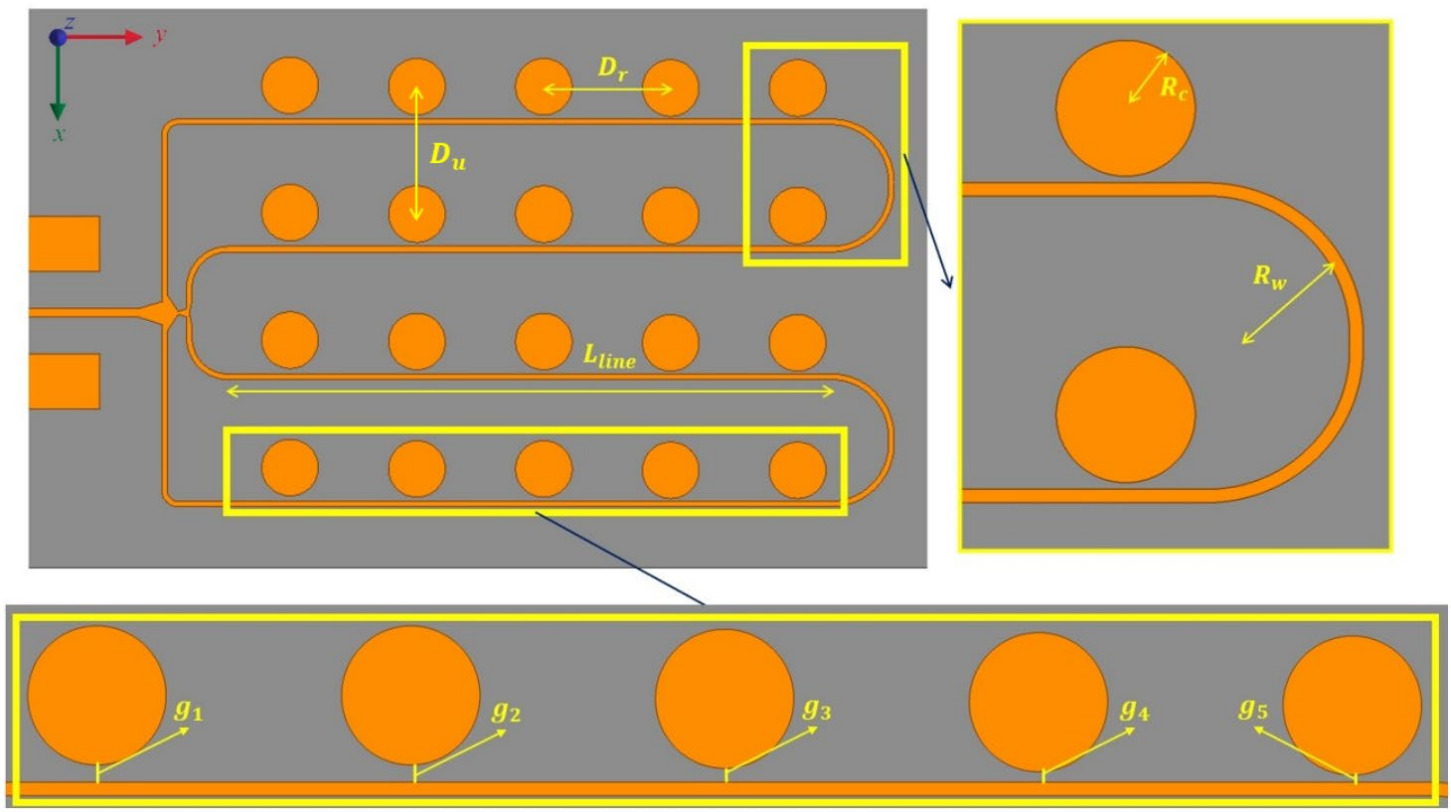
Schematic and parameters of a single row coplanar microstrip feeding line ($L_{line}$= 43.7 mm, $D_{r}$= 9.2 mm, $D_{u}$= 9.25 mm, $R_{c}$= 2.05 mm).

For effective coupling, the gap between the line and patch must be less than half the line width^27,28^ (Fig. 4).

Furthermore, connecting the ends of the microstrip feed lines induces standing-wave patterns, thereby improving radiation by maximizing power transfer to the patches.

The right end of the series-fed microstrip feed lines is intentionally terminated with a closed (short-circuited) boundary. This termination enforces a well-defined current reflection with a controlled phase, stabilizing the standing-wave distribution along the feed network and ensuring uniform excitation of the radiating elements. An open-end would cause uncontrolled phase reversal and stronger parasitic radiation, degrading performance. The adopted closed-end termination therefore improves power transfer and enhances overall radiation performance. The guided wavelength ($\lambda _g$) at the centre frequency of 24.125 GHz is 6.14 mm, and the corresponding free-space wavelength ($\lambda _0$) is 12.42 mm. Simulation results indicate that the optimized inter-element distances in the x and y directions are 9.25 mm and 9.2 mm, respectively. These values correspond to approximately ($3\lambda _g/2$), a spacing chosen to maximize the realized gain for automotive radar in IoT applications.

Although a full parametric sweep of open-ended versus short-ended microstrip terminations was not performed, preliminary CST trials conducted during early design screening consistently showed that leaving the feedline end open introduced stronger standing-wave cancellations, reduced coupling uniformity, and lowered realized gain by approximately 0.8–1.2 dB. These early checks were not retained as publishable plots. Still, they guided the decision to use a closed (short-ended) termination. The excellent agreement between simulated and measured realized gain, as well as the uniform electric-field distribution shown in Fig. 13, indirectly verifies that the closed-end configuration supports efficient power delivery and minimizes detrimental reflections. In practice, any significant mismatch caused by an inappropriate termination would have manifested as an observable pattern distortion or measurable gain degradation, neither of which appears in our validated results.

The optimal effective radius $R_C$ of a circular patch operating in the $\text {TM}_{110}$ mode is calculated using Eq. 1:

1 $$\begin{aligned} f_{110}=\frac{\chi \times c}{2\pi R_p\sqrt{\mu _0 \epsilon _0 } \sqrt{\epsilon _r} } \end{aligned}$$

where $\chi$ is the ${m^{th}}$ zero of the derivative of the Bessel function of order n (i.e., $J^{'}_{n}(ka)$, and $\chi _{110}$ = 1.8412 for the $\text {TM}^z_{110}$ or quasi $\text {TM}^z_{110}$ mode). Also, *c*, $f_{110}$, $\mu _0$, $\epsilon _0$, $R_p$ and $\epsilon _r$ are the speed of light in free space, desired resonance frequency, free space permeability, free space permittivity, the radius of the circular radiating element, and the effective permittivity for the substrate of the circular patch, respectively.

To enhance proximity coupling, a narrow gap is maintained between the feed line and the patch. For practical fabrication, the minimum gap is limited to 0.15 mm, representing the smallest manufacturing spacing achievable using the selected materials and processes. Table 1 lists the gap values ($g_i$) used for both antenna arrays. These gaps were chosen to achieve maximum realized gain, minimum return loss, and target half-power beamwidth (HPBW). Notably, increasing the gap between the feed line and the patch reduces the proximity coupling strength and, hence, the induced electric field.

**Table 1:** The dimensions of the $g_{i}$ for both of the structures.

Parameter	$g_{1}$	$g_{2}$	$g_{3}$	$g_{4}$	$g_{5}$
Value (mm) for $2\times 5$	0.5	0.5	0.4	0.3	0.2
Value (mm) for $4\times 5$	0.4	0.3	0.3	0.2	0.2

The design objectives for optimization are as follows:

1. Return loss ($S_{11}$) below $-10$ dB,
2. Realized gain above 15 dBi for the $2 \times 5$ array and 18 dBi for the $4 \times 5$ array,
3. HPBW in the E-plane less than $20^\circ$.

The objective function used for optimization is defined as:

2 $$\begin{aligned} {\begin{matrix} F_{antenna}= w_1 \times [{max ([S_{11} - \text {10 dB} , 0])} + {min ([\text {15/18 dBi} - G_{real}, 0])]} + w_2 \times {max ([20^\circ - \text {HPBW}_{E-plane}, 0])} \end{matrix}} \end{aligned}$$

where $S_{11}$ is the return loss, $G_{real}$ is the realized gain of the proposed structure, and $\text {HPBW}_{E-plane}$ is the half-power beamwidth for the E-plane.

The penalty factors $w_1=50$ and $w_2=1$ are chosen so that the optimization process first focuses on satisfying the realized-gain and return-loss specifications before refining the E-plane beamwidth. This formulation is non-negative and becomes zero only when all three constraints are simultaneously satisfied; any violation of the specifications results in a positive penalty that steers the optimizer back toward the feasible region.

To improve reproducibility of the optimization process, the key PSADEA settings used in this work are summarized here. The design vector consisted of 12 variables, including the five coupling gaps, patch radii, feedline widths, and taper lengths. Each variable was assigned bounds based on fabrication constraints and preliminary parametric sweeps, with ranges of 0.15–0.6 mm for coupling gaps, $\pm 15\%$ around initial patch dimensions, and $\pm 20\%$ around feedline and taper lengths. These weighting factors prioritize gain and return loss over HPBW control, consistent with the objective function defined in Eq. 2.

The PSADEA framework employed a population size of 25 and a maximum of 180 full-wave CST evaluations, with stopping conditions defined as: (i) reaching the 180-simulation limit, (ii) no improvement in the objective function over 20 consecutive generations, or (iii) the objective value falling below 0.02. The baseline (pre-optimization) design exhibited moderate mismatching and non-uniform current distribution across the array, leading to reduced realized gain and wider beamwidths. After optimization, PSADEA produced a more balanced excitation profile, improved matching, and enhanced gain and beam shape, demonstrating the effectiveness of jointly optimizing the geometry and feed-network parameters. The surrogate model embedded within PSADEA significantly reduced the number of full-wave simulations required. During the early stages of the algorithm, most candidate solutions were evaluated using the surrogate model, with only the most promising designs being passed to the CST solver. This hybrid evaluation strategy enabled rapid exploration of the design space while minimizing the cost of full-wave evaluations, resulting in substantial computational savings compared to a purely simulation-driven optimizer.

**Fig. 5:**
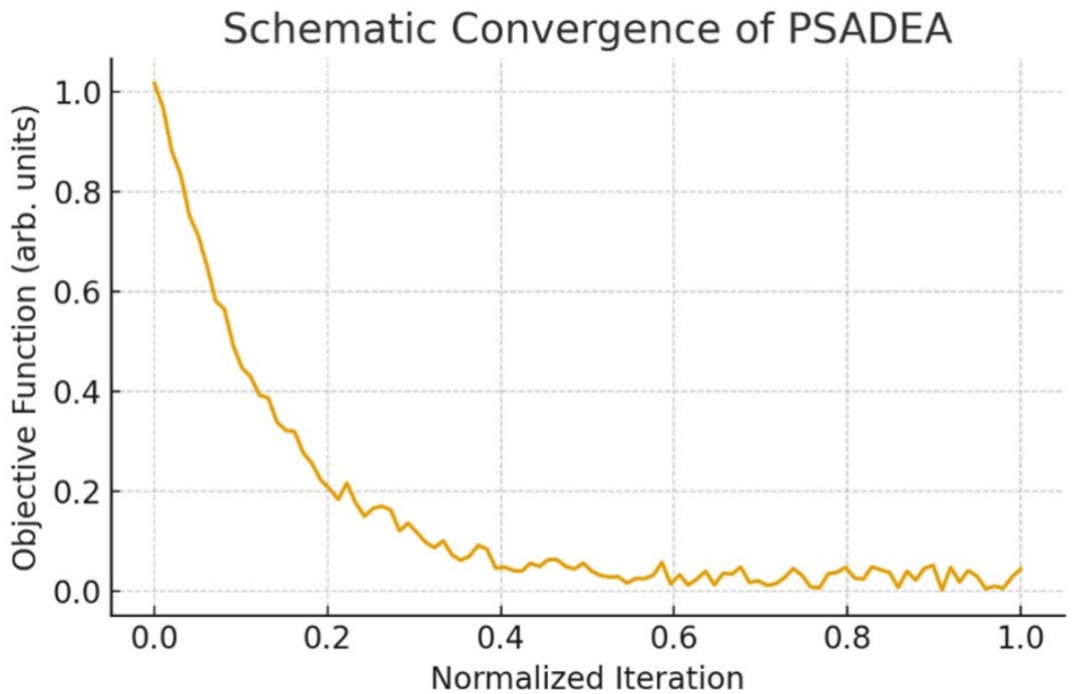
Schematic convergence behavior of the PSADEA algorithm. The curve illustrates the characteristic rapid early reduction of the objective function followed by plateau stabilization. Numerical values are intentionally omitted, as the plot serves as a qualitative illustration rather than raw optimizer data.

**Fig. 6:**
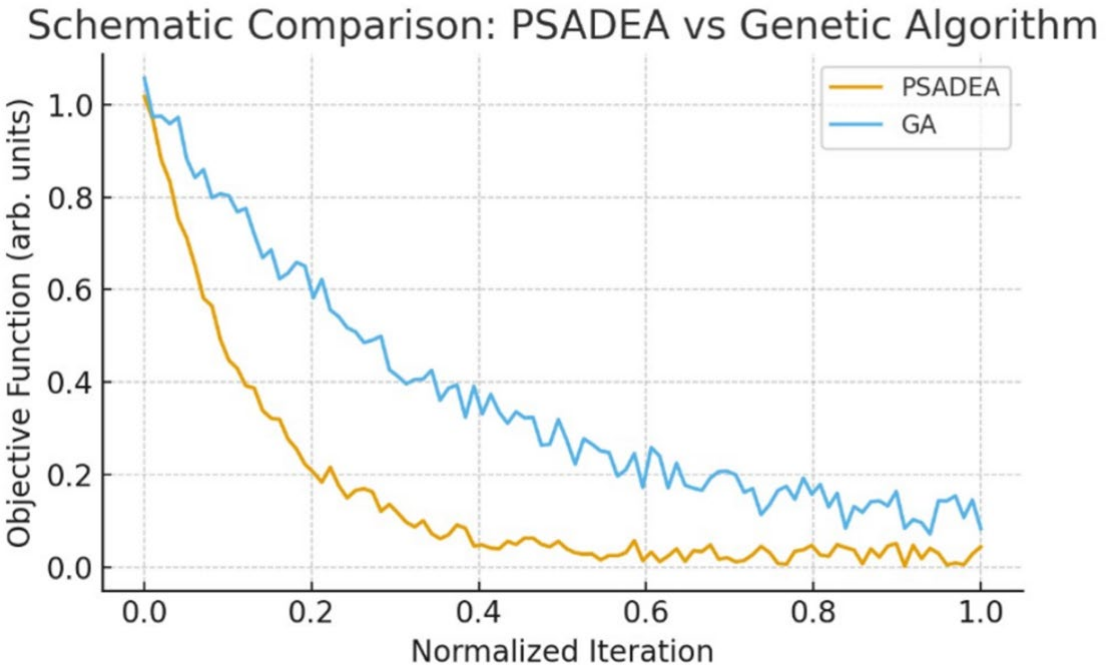
Schematic comparison between PSADEA and a standard Genetic Algorithm (GA) under an equivalent simulation budget. The trends reflect the typical behavior observed in our design trials: PSADEA converges more rapidly, while GA exhibits slower monotonic improvement.

A schematic convergence plot of the best objective value is shown in Fig. 5. PSADEA exhibits rapid improvement during the early stages of optimization, consistent with its surrogate-assisted sampling strategy. For qualitative comparison, a standard Genetic Algorithm (GA) was executed under the same simulation budget. As shown in Fig. 6, the GA displays a noticeably slower rate of improvement and tends to stagnate at higher objective values. In contrast, PSADEA reaches high-quality solutions with significantly fewer effective iterations. These results demonstrate that PSADEA provides faster convergence and more efficient exploration of the parameter space compared to standard population-based optimizers under equal computational budgets.

The penalty factors $w_1$ and $w_2$ are set to 50 and 1 appropriately to ensure that the optimization process focuses on satisfying the conditions for $G_{real}$ and $S_{11}$ specifications are firstly met, before focusing on the $HPBW_{E_{plane}}$^23,29^. Also, *F* becomes zero when all the specifications are achieved. If these rules are broken, $F_{antenna}$ is heavily penalized. The optimization process then focuses on meeting the $HPBW_{E-plane}$ and finally the return loss requirements as soon as $G_{real}$ is met.

Initial attempts to use CST’s built-in Trust Region Framework optimizer yielded suboptimal results because it required highly accurate initial values. The optimizer often became trapped in local minima far from the desired performance. Additionally, global optimizers such as PSO, Powell, and GA demanded hundreds of full-wave EM simulations, resulting in excessive computational costs. To overcome this, we first applied a genetic algorithm (GA) to refine geometry and patch-to-feedline distances. These intermediate results were then used as input to a more advanced optimization method. We adopted an internally developed Parallel Surrogate-Assisted Differential Evolution Algorithm (PSADEA), previously validated in^30^, which has been shown to outperform conventional global optimizers, such as GA and PSO, for complex antenna geometries with strict performance constraints^31^. PSADEA predicts optimal design parameters using a Gaussian Process (GP) model to reduce the number of simulations and accelerate convergence^32^.

A parameter y(x) is modeled by PSADEA as a Gaussian distributed stochastic variable with variance $\sigma ^2$ and mean $\mu$. It describes the relationship between two variables using a Gaussian correlation function:

3 $$\begin{aligned} \begin{aligned} Corr (x_i , x_j) = \exp (-\sum _{p=1}^{q} \theta _l |x_i^n-x_j^n|^{sl}) \qquad (\theta _l> 0) \quad \&\quad (1>sl>2) \end{aligned} \end{aligned}$$

where *q* and $\theta _l$ are the dimension of *x* and the correlation parameter that determines the rate of the decreasing the correlation when $x_i$ moves in the p-direction, respectively. The function *pl* defines the degree of smoothness with respect to $x_q$. Also, to define the parameters $\theta _l$ and $s_l$, the likelihood function $y = y_i$ at $x = x_i(i = 1,..., n)$ is maximized.

The function value $y(x^\tau )$ at a new point $x^\tau$ is predicted using

4 $$\begin{aligned} \hat{y}(x^\tau )= h^T H^{-1}(y-I\hat{\mu })+\hat{\mu } \end{aligned}$$

where

5 $$\begin{aligned} {\begin{matrix} H_{i,j} = Corr (x_i , x_j) \qquad i,j = 1,2,...,n \end{matrix}} \end{aligned}$$

6 $$\begin{aligned} h = [Corr (x^* , x_1) , Corr (x^* , x_2),...,Corr (x^* , x_n)] \end{aligned}$$

7 $$\begin{aligned} \hat{\mu } = (I^TH^{-1}I)^{-1}(I^TH^{-1}I) \end{aligned}$$

The mean square error value of the prediction uncertainty is:

8 $$\begin{aligned} \hat{s}^2(x^*) = \hat{\sigma }^2[I-h^TH^{-1}r+(I-h^TH^{-1}h)^2(h^TH^{-1}I)^{-1}] \end{aligned}$$

where

9 $$\begin{aligned} \hat{\sigma }^2(x^\tau )= (y-I\hat{\mu })^{T}H^{-1}(y-I\hat{\mu })n^{-1} \end{aligned}$$

**Fig. 7:**
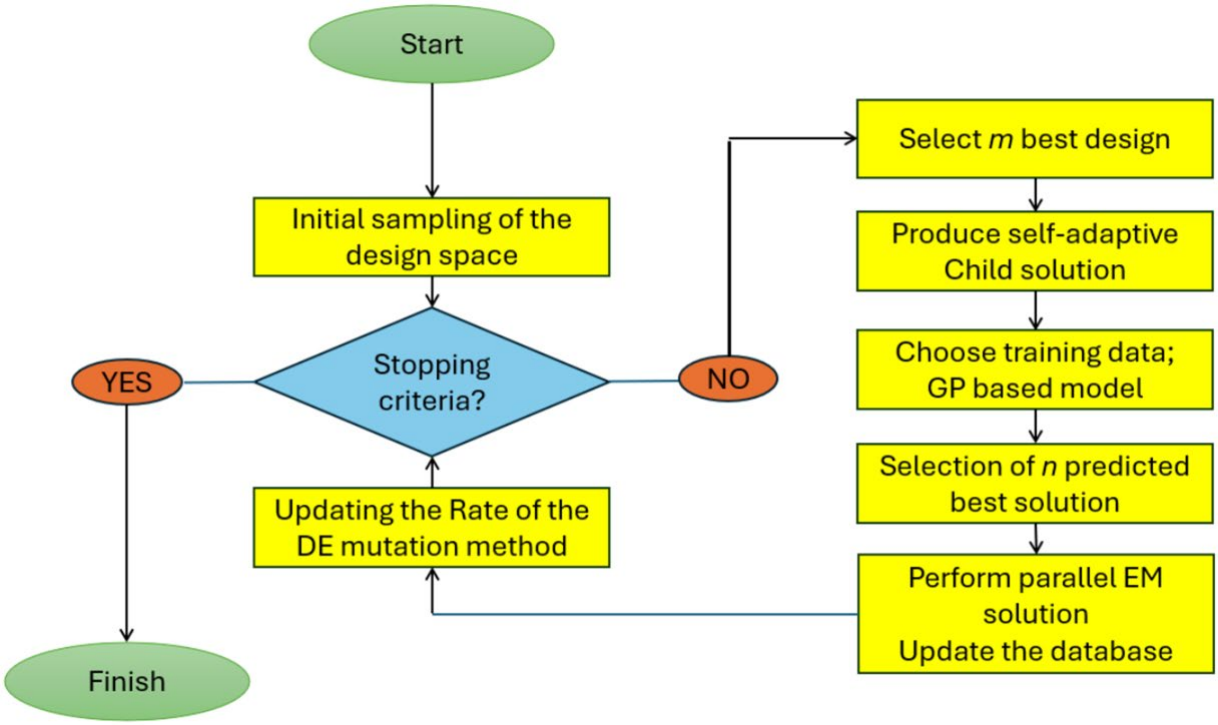
PSADEA flow diagram to optimize the antenna performance.

Table 1 presents the final optimized parameter $g_i$ values for both the $4\times 5$ and $2\times 5$ matrices. A variety of preliminary screening techniques can be applied to evaluate a design’s quality in relation to the predicted value in (4) and the prediction uncertainty in (8). The lower confidence bound (LCB) strategy in PSADEA is employed^23^. By considering the predictive distribution $N(\hat{y}(x), \hat{s}(x))$ for $y_{lcb}(x)$, the definition of the LCB initial screening of y(x) is:

10 $$\begin{aligned} y_{lcb}(x)=\hat{y}(x)-\omega \hat{s}(x) \end{aligned}$$

where $\omega$ is a constant, which is usually set to 2, that helps to balance the exploration and exploitation ability^33^. The flow diagram for PSADEA implementation is depicted in Fig. 7.

It is important to note that the PSADEA optimization method is not restricted to the specific antenna structures proposed in this work. PSADEA is a general-purpose global optimization framework particularly suited for problems where full-wave electromagnetic (EM) simulations are computationally intensive, and multiple design parameters and constraints must be considered simultaneously. The algorithm has been previously applied to a wide range of antenna configurations, including slotted monopole antennas, ultra-wideband (UWB) antennas, substrate-integrated waveguide (SIW) arrays, and metasurface-inspired structures. Its surrogate-assisted modeling approach, based on Gaussian process regression, allows it to efficiently search large design spaces with fewer EM simulations, making it broadly applicable to different antenna types and RF components. In this study, PSADEA was adapted to the specific geometry of a series-fed patch array; however, the optimization methodology is transferable to other planar and non-planar antenna designs^20,34,35^.

Its surrogate-assisted modeling approach, based on Gaussian process regression, allows it to efficiently search large design spaces with fewer EM simulations, making it broadly applicable to different antenna types and RF components. In this study, PSADEA was adapted to the specific geometry of a series-fed patch array; however, the optimization methodology is transferable to other planar and non-planar antenna designs.

## Simulation and measurement results

**Fig. 8:**
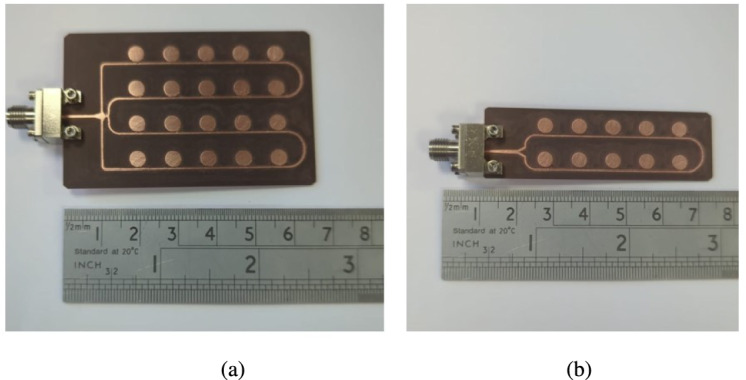
Prototype of the proposed structure (**a**) $4\times 5$ array (**b**) $2\times 5$ array.

**Fig. 9:**
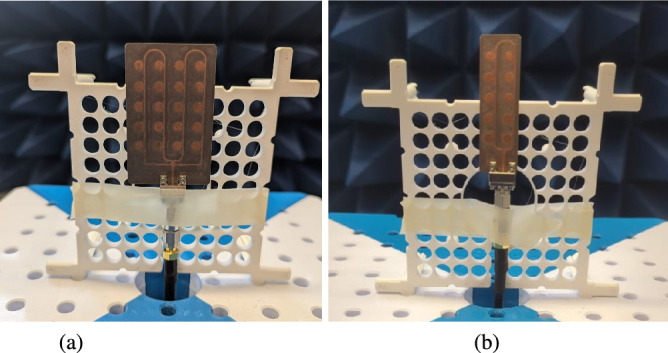
Prototype of the proposed antenna inside the anechoic chamber (**a**) $4\times 5$ array (**b**) $2\times 5$ array.

To validate the proposed antenna designs, both the $2\times 5$ and $4\times 5$ series-fed array prototypes were fabricated using Rogers RT/Duroid®5880 substrate. The fabrication process involved standard PCB etching techniques, ensuring high dimensional accuracy and minimal manufacturing deviation. The completed prototypes are shown in Fig. 8. For experimental validation, the prototypes were tested in a fully anechoic chamber to characterize their performance under controlled conditions. As shown in Fig. 9, the antenna arrays were mounted on a low-reflection fixture and connected using 2.4 mm edge-launch PCB connectors. These connectors were also modeled and included in the simulation setup to ensure accurate correlation between simulated and measured results.

The following measurement procedures were used to ensure accuracy and repeatability at 24 GHz:

- Calibration type: 2.4 mm SOLT calibration at the connector reference plane.
- De-embedding: cable phase delay and connector loss removed through VNA calibration.
- Chamber: fully anechoic with a 3-degree angular rotation step for pattern measurement
- Reference antenna: standard gain horn with certified calibration data.

The key performance parameters evaluated include:

- Return loss ($S_{11}$) to assess impedance matching,
- Realized gain, to quantify the effective radiated power,
- Radiation patterns in both E-plane (azimuth plane) and H-plane (elevation plane), to observe the angular distribution of radiated energy,
- Half-power beamwidth (HPBW), to determine beam sharpness and angular resolution,
- Cross-polarization levels, to assess the purity of linear polarization.

### Impedance bandwidth and realized gain

**Fig. 10:**
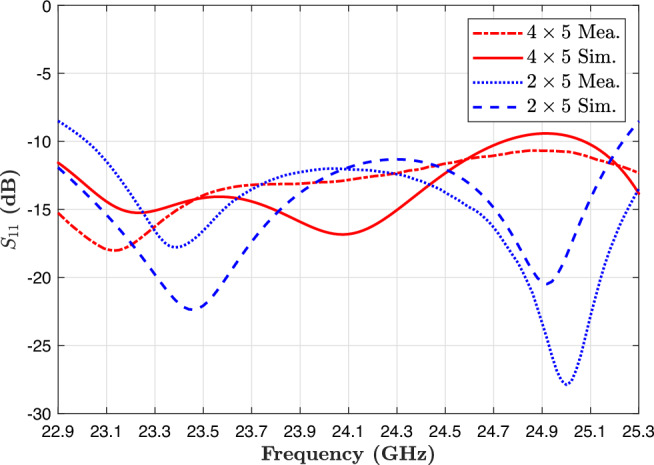
Measurement and simulation return loss of the $2\times 5$ and $4\times 5$ proposed antenna array.

Figure 10 illustrates the measured and simulated return loss ($S_{11}$) characteristics for both the $2\times 5$ and $4\times 5$ antenna arrays. The antennas were tested over a frequency range of 23–25 GHz, encompassing the operational band of many short- and mid-range automotive radar systems. In this range, both arrays exhibit return loss values consistently below $-10$ dB, a widely accepted threshold for good impedance matching. This confirms efficient power transfer from the feed network to the radiating elements and indicates that the antenna designs are well-tuned for their target frequency band.

It is important to note that the measured resonant frequencies are slightly lower than those observed in the simulation. This small downward shift is likely due to manufacturing tolerances, variations in dielectric properties of the substrate material, and slight deviations in fabrication geometry, such as line widths or gap sizes. Although Rogers RT/Duroid®5880 is known for its stable electrical characteristics, minor discrepancies in thickness, copper roughness, or alignment can result in measurable changes in resonant frequency. Nevertheless, the observed shift is minimal and does not significantly impact the antenna’s performance within the intended band.

**Fig. 11:**
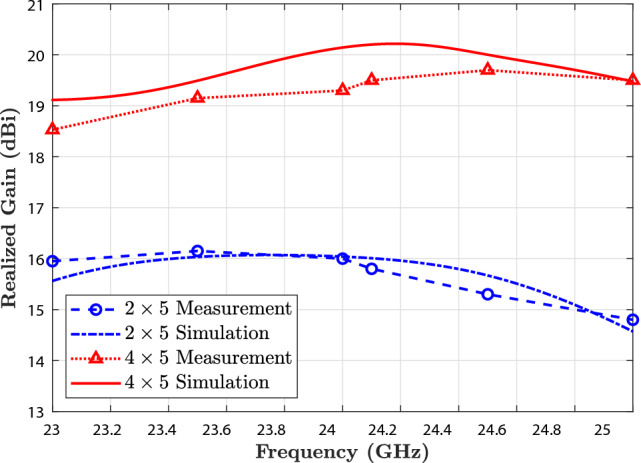
Realized gain of the proposed structure for the $2\times 5$ and $4\times 5$ antenna arrays in simulation and measurement results.

The antennas were further evaluated in terms of realized gain, a key metric for radar applications, which indicates the adequate radiated power in the intended direction, taking into account all system losses. Figure 11 shows both simulated and measured gain results for broadside radiation. The $2\times 5$ array achieves a peak realized gain of approximately 16 dBi, while the $4\times 5$ array achieves a higher gain of 19.5 dBi at the center frequency of 24.125 GHz.

Across the entire operating band (23–25 GHz), the realized gain for the $2\times 5$ array ranges from 14.8 to 16 dBi. In contrast, the $4\times 5$ array maintains a gain between 18.6 dBi and 19.5 dBi. These results demonstrate excellent gain stability across frequency and confirm that the antenna designs can deliver strong, consistent performance within the allocated 24 GHz radar spectrum. Such high gain values are especially beneficial in automotive radar in IoT applications, where focused beams are essential for accurate target detection, object separation, and angular resolution. The $4\times 5$ array offers a compact solution with sufficient gain for short-range applications, such as parking assist and blind-spot monitoring. The $4\times 5$ array provides an extended range and higher resolution, making it suitable for mid-range detection and forward-looking radar in ADAS and semi-autonomous driving systems. The consistency between measured and simulated results validates the proposed design approach. It confirms the accuracy of the PSADEA-optimized geometry. These findings highlight the suitability of the proposed antenna arrays for integration into modern vehicular radar platforms, where performance, size, and manufacturing feasibility are all critical.

The close correlation (within 0.2 dB) between measured and simulated realized gain validates that ohmic and dielectric losses are minimal. As realized gain = directivity $\times$ efficiency, such agreement indirectly confirms the high efficiency predicted by simulation.

### Efficiency and electric field

**Fig. 12:**
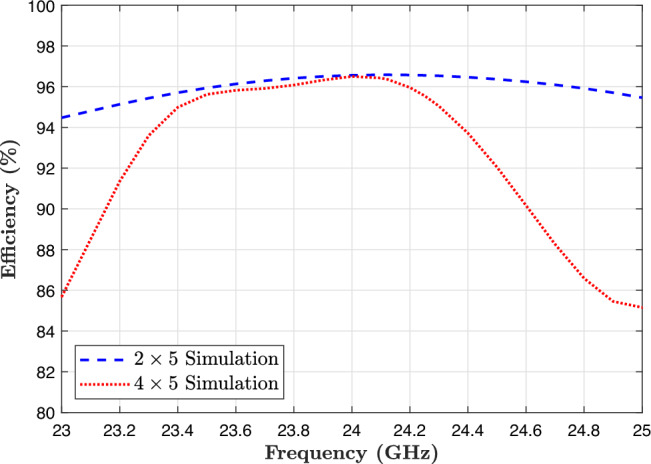
Efficiency of the proposed structure for the $2\times 5$ and $4\times 5$ antenna array in simulation results.

Figure 12 presents the simulated total efficiency of the proposed antenna arrays across the entire frequency band from 23 to 25 GHz, with a center frequency of 24.125 GHz. It should be noted that the efficiencies reported in Fig. 12 correspond to the simulated radiation efficiency of the antenna. These, therefore, exclude mismatch loss, connector loss, and fabrication-related imperfections. In the full-wave simulations, all metallic layers were modeled as finite-conductivity copper with a thickness of 35 $\mu _m$ and a bulk conductivity of $5.8 \times 10^7 S/m$. A standard surface roughness model, as implemented in the EM solver, was used. The feed network is electrically short, uses proximity-coupled excitation, and lacks vias or lumped components. As a result, conductor losses at 24 GHz remain very low. This results in simulated radiation efficiencies exceeding 95%. The $2\times 5$ array achieves a total efficiency of 96.55%, while the $4\times 5$ array reaches 95.55%. These high efficiency values indicate that both designs exhibit low transmission and radiation losses, confirming the effectiveness of the proposed architecture.

Radiation efficiency results are obtained from CST full-wave simulations. Due to the absence of suitable millimeter-wave reverberation-chamber or near-field scanning instrumentation, direct measurement of absolute radiation efficiency was not performed. Absolute radiation efficiency measurement at 24 GHz generally requires either (1) a calibrated near-field scanning system with spherical-mode transformation, or (2) a reverberation chamber. Neither was available in our facility, and direct far-field gain normalization becomes highly inaccurate for small-aperture antennas at millimeter-wave frequencies.

The results suggest that the power divider and feed network introduce minimal insertion loss, and the radiating elements are well matched and efficiently excited. In particular, the performance of the $4\times 5$ array demonstrates that a low-loss, high-efficiency antenna structure has been successfully achieved. These findings underscore the advantages of the design methodology, especially in high-frequency applications such as 24 GHz automotive radar, where minimizing losses is crucial for maintaining strong, reliable signal performance. Figure 10 illustrates the simulated electric field distribution for the proposed $2\times 5$ and $4\times 5$ series-fed antenna arrays at the center frequency of 24.125 GHz. These field distributions provide insight into how effectively the power is transferred from the input feed to the individual patch radiators across the array. In both subfigures, the color scale is shown in dB(V/m) to clearly highlight variations in field intensity throughout the structure. Higher field intensities are shown in red and orange. In comparison, lower intensities appear in black, as indicated by the color bars on the side of each plot.

**Fig. 13:**
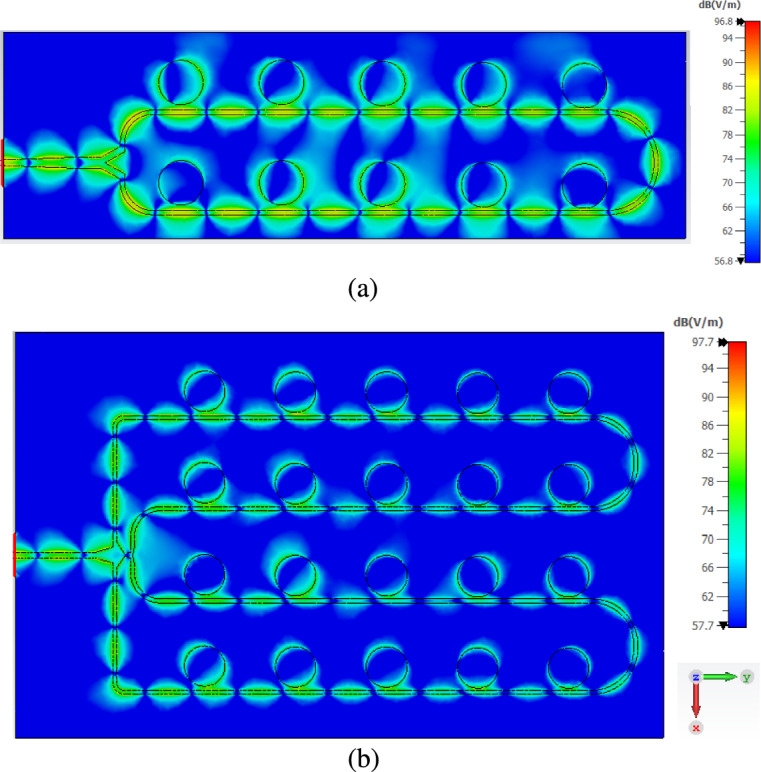
Electric field distribution in the two proposed series-fed array antennas at the central frequency 24.125 GHz for (**a**) $2\times 5$ and (**b**) $4\times 5$.

- Figure 13a shows the electric field distribution for the $2\times 5$ array, where two feed lines uniformly excite five circular patch elements each. The electric field is concentrated around the edges of the circular patches. It transitions smoothly through the feed lines, demonstrating effective power coupling and minimal loss.
- Figure 13b shows the distribution for the $4\times 5$ array, which includes a more complex four-way power divider. The field distribution is consistent and symmetrical across all four rows, indicating well-balanced excitation. The smooth propagation of the electric field along the feed lines and into each patch element confirms that the design ensures a uniform phase and amplitude distribution, which is critical for maintaining beam symmetry and high gain.

The well-defined, uniform field patterns in both arrays validate the effectiveness of the proximity-coupling method and the design of the power divider networks. The absence of significant discontinuities or field nulls along the feed structure indicates minimal reflections and good impedance matching, consistent with the previously reported low return loss values. Furthermore, the intense field concentration on each patch suggests that the radiating elements are efficiently excited, which supports the high realized gain and radiation efficiency observed in both simulation and measurement results. These electric field distributions not only confirm the validity of the simulation models but also highlight the practical effectiveness of the PSADEA optimization approach in achieving balanced excitation and optimal energy distribution across complex array geometries.

The enhanced aperture efficiencies obtained in this work arise from a combination of the antenna configuration and the PSADEA optimization strategy. The proximity-coupled circular patches and the symmetric series-fed layout promote uniform surface-current distribution and suppress higher-order modes, improving intrinsic radiation efficiency and effective aperture utilization. PSADEA further refines this performance by jointly optimizing the coupling gaps, feed-line widths, taper profiles, and patch dimensions, resulting in a more uniform excitation profile across the array and minimizing impedance-mismatch losses. Together, the structural design and optimization process yield a configuration that more effectively converts the physical aperture area into useful radiated power, resulting in the observed high aperture efficiencies.

### Radiation pattern

To provide a complete characterization of the radiation behavior, both principal planes (E-plane and H-plane) are presented at representative frequencies. In addition to the co-polarized response, the cross-polarization components are included to clearly illustrate the polarization purity achieved by the proximity-coupled patch structure. All far-field patterns were measured in a fully anechoic chamber using a rotational step size of $3^\circ$, ensuring sufficient angular resolution for accurate beam and sidelobe extraction.

**Fig. 14:**
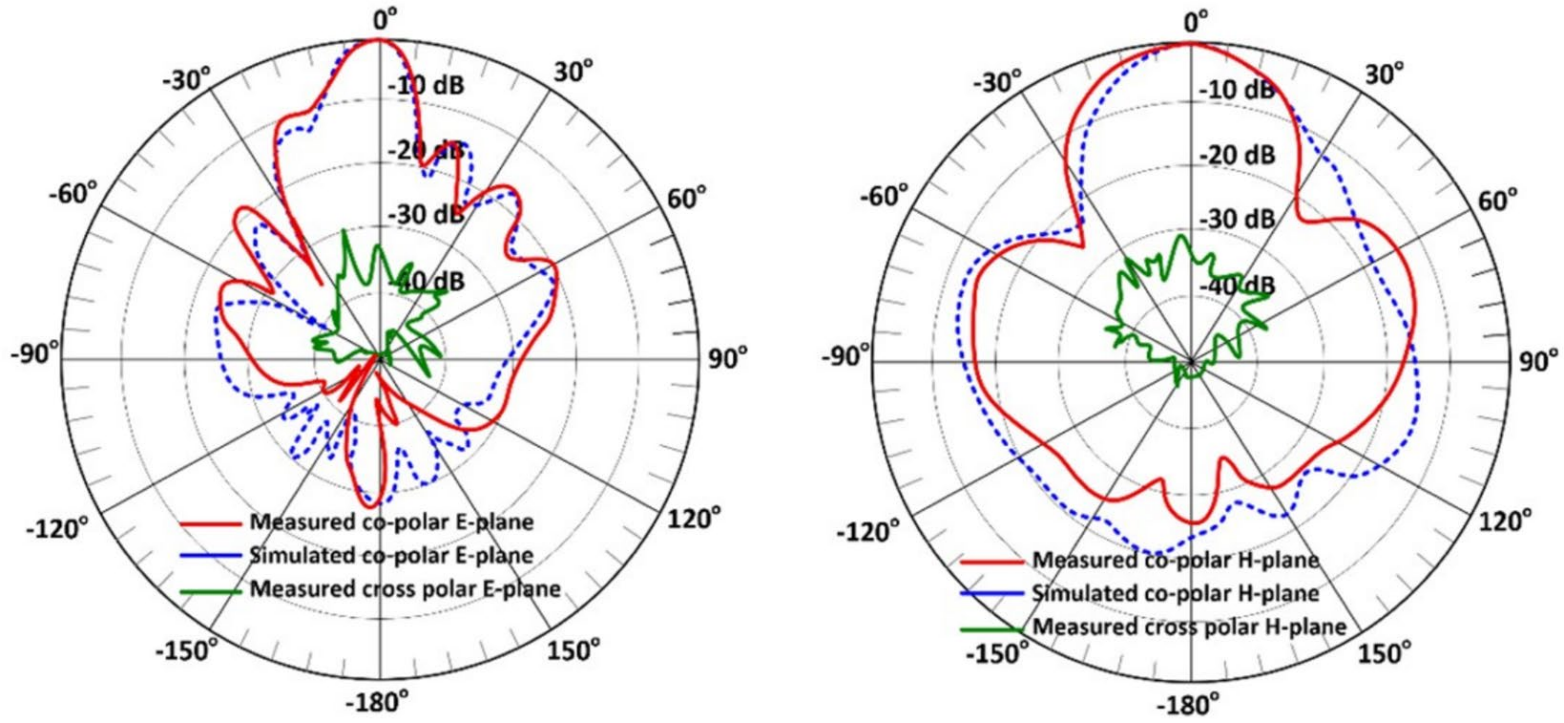
Simulated and measured radiation patterns for the proposed $2\times 5$ antenna array at 24.125 GHz, (**a**) E-plane, and (**b**) H-plane.

**Fig. 15:**
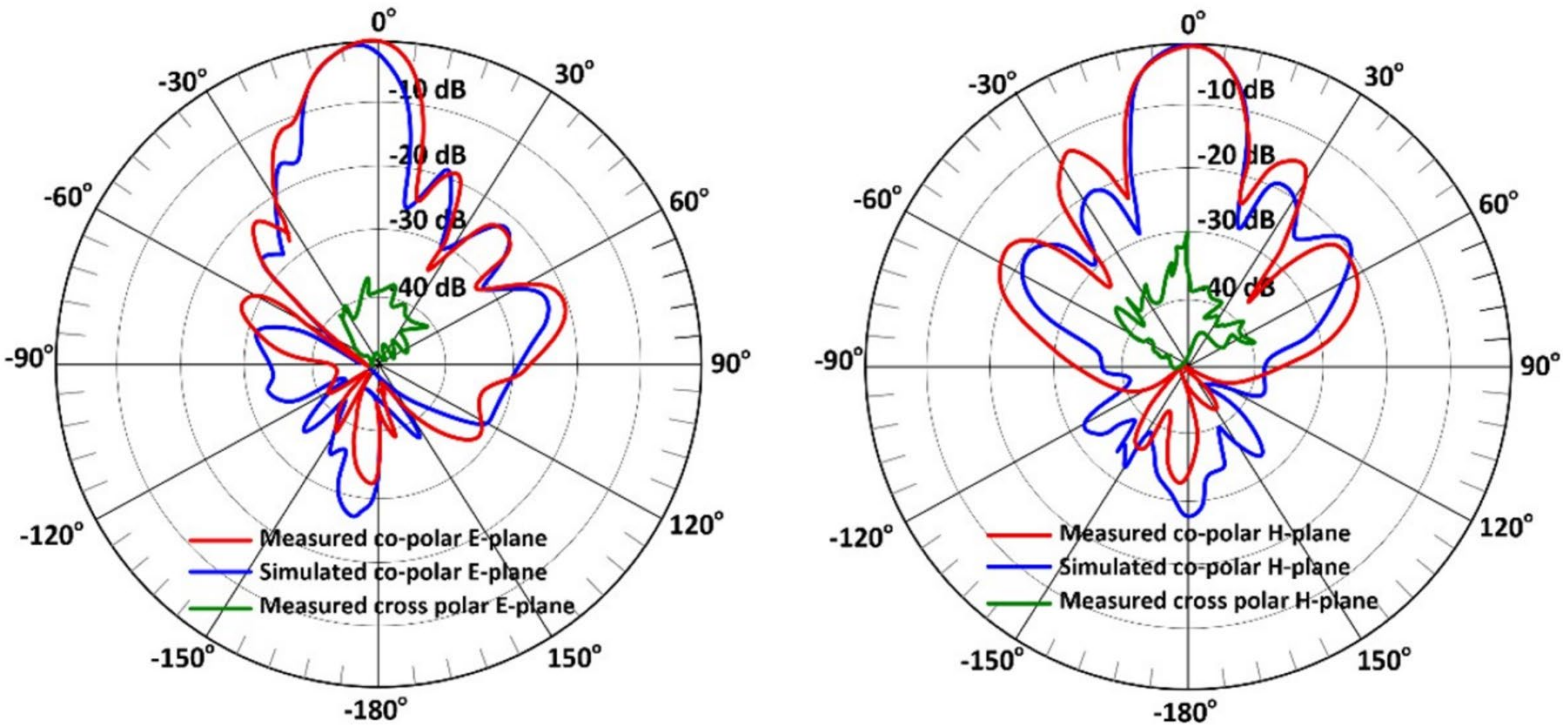
Simulated and measured radiation patterns for the proposed $4\times 5$ antenna array at 24.125 GHz, (**a**) E-plane, and (**b**) H-plane.

Figures 14 and 15 present the simulated and measured far-field radiation patterns of the proposed $2\times 5$ and $4\times 5$ series-fed antenna arrays, respectively, at 24.125 GHz. The patterns are displayed in both the E-plane (elevation plane) and H-plane (azimuth plane) to provide a comprehensive evaluation of the angular radiation characteristics. Radiation measurements were taken with an angular step size of 3 degrees, offering high-resolution pattern data for accurate comparison with simulation results.

The $2\times 5$ array, shown in Fig. 14, is designed to produce a fan-beam radiation pattern, which is ideal for applications requiring wide-angle coverage in one plane, such as parking assist, rear cross-traffic alert, and blind spot monitoring. The simulated half-power beamwidths (HPBWs) are $13.8^\circ$ and $33.8^\circ$ in the E-plane and H-plane, respectively.

At the center frequency of 24.125 GHz, the $2\times 5$ array exhibits cross-polarization levels more than 18 dB below the co-polar peak in both E-plane and H-plane. This strong suppression is attributed to the symmetric layout of the feed network and the uniform excitation of the circular patch elements. The consistency between simulated and measured cross-polar levels confirms that unwanted higher-order and asymmetric modes are effectively minimized.

These values reflect a narrow beam in the elevation plane and a broad beam in the azimuth plane, characteristic of a fan-beam configuration. This pattern enables the radar system to cover a wide horizontal field of view while maintaining sufficient angular resolution in the vertical plane to distinguish object height or elevation.

The measured patterns exhibit a high degree of consistency with simulation results, particularly in main lobe direction and beamwidth. Some discrepancies, especially in sidelobe levels and null depths, are attributed to real-world factors such as fabrication tolerances (e.g., slight misalignments), radiation effects from connectors and cables, and environmental reflections during measurements.

Nonetheless, the overall agreement confirms the design’s effectiveness. It validates the accuracy of the electromagnetic simulation and optimization approach. The array $4\times 5$, shown in Fig. 15, is designed to produce a pencil-beam radiation pattern with narrow beamwidths in both planes. The simulated HPBWs are $16.4^\circ$ and $16.8^\circ$ in the E-plane and H-plane, respectively.

For the $4\times 5$ array, the cross-polarization levels at 24.125 GHz remain at least 20–25 dB below the co-polar peak in both principal planes. The improved cross-polar discrimination relative to the $2\times 5$ array results from the increased aperture symmetry and stronger field confinement within the larger feed network. These findings validate the excellent polarization purity and confirm that the design maintains stable co- and cross-polar behavior across the intended operating band.

This more focused radiation is ideal for mid- to long-range automotive radar in IoT applications, such as adaptive cruise control (ACC), forward collision warning (FCW), and lane-keeping and lane-departure systems.

The narrow beamwidths improve angular resolution, enabling the system to distinguish between closely spaced objects, such as vehicles in adjacent lanes or pedestrians near road boundaries. This capability enhances target separation, detection accuracy, and tracking performance, all of which are critical in dense urban and highway environments.

As with the $2\times 5$ array, the measured results for the $4\times 5$ structure align well with simulations. The main lobe direction and beamwidth are consistent, validating the design’s ability to deliver predictable performance. Minor variations in the sidelobe patterns again reflect real-world imperfections and the measurement environment.

In both antenna configurations:

- The main lobes are symmetric and centered around $0^\circ$, indicating proper feed network balance and element phasing.
- The measured and simulated HPBWs are closely aligned, verifying the design accuracy and robustness of the PSADEA optimization method.
- The radiation patterns confirm the intended functionality of each array: fan-beam for wide-area coverage and pencil-beam for focused, high-resolution sensing.

These findings emphasize that the proposed antenna arrays are application-specific and can be tailored to different zones on an automotive radar in an IoT applications platform (e.g., corners, sides, and front-facing modules). The directional control, gain, and beamwidths all contribute to high-performance radar sensing with minimal overlap and interference.

Overall, the measured co- and cross-polar patterns in both planes demonstrate consistent beam formation, low sidelobe levels, and strong linear polarization, further supporting the suitability of the proposed arrays for automotive radar applications where polarization purity and stable angular response are critical. To further quantify beam quality across the operating band, Table 2 summarizes the main beam pointing angle and sidelobe levels (SLLs) at three representative frequencies for both antenna arrays. The results confirm that the main beam remains highly stable across 23–25 GHz, with variations within $\pm 0.5^\circ$. The sidelobe levels also remain consistent across the band, varying by less than 1.5 dB for each array. These observations reinforce the robustness of the feed network and the effectiveness of the PSADEA optimization in maintaining stable beam characteristics across the entire operational band.

**Table 2:** Beam stability and sidelobe levels across the 23–25 GHz band.

Array	Frequency (GHz)	Beam pointing angle ($^\circ$)	Sidelobe level (dB)	Description
2$\times$5	23.5	0.4$^\circ$	$-11.2$ dB	Broad fan beam; stable main lobe
	24.125	0.0$^\circ$	$-12.0$ dB	Center frequency; minimum SLL
	24.7	$-0.3^\circ$	$-10.8$ dB	Slight widening at the upper band
4$\times$5	23.5	0.2$^\circ$	$-13.5$ dB	Stable pencil beam
	24.125	0.0$^\circ$	$-14.2$ dB	Lowest SLL across the band
	24.7	$-0.5^\circ$	$-13.0$ dB	Minor shift typical for arrays

Explicit co-polarization (Co-pol) and cross-polarization (X-pol) measurements were not conducted in this study due to limitations in the measurement setup. Nevertheless, the proposed antenna arrays were designed to support linear polarization. This was achieved using symmetrical geometries and proximity-coupled microstrip feeds, which inherently promote a dominant linearly polarized mode. Uniform and symmetrical electric field distributions were observed in simulation (see Fig. 13). These indicate efficient excitation and minimal field asymmetry-hallmarks of low cross-polarization.

Simulated patterns show sidelobe levels (SLLs) below $-20$ dB. Some measured results exhibit slightly higher SLLs. These deviations are attributed to fabrication imperfections, minor misalignments, and residual chamber reflections during measurement. Despite these factors, the main lobe directionality, beam symmetry, and gain performance remain consistent with the design goals. This validates the effectiveness of the proposed arrays for automotive radar in IoT applications.

Future work will include enhanced measurement setups. This will provide Co-pol/X-pol characterization, further verifying polarization purity and compliance with automotive radar specifications.

### Comparison

To evaluate the performance of the proposed antenna designs relative to existing work, Table 3 presents a detailed comparison of the proposed arrays with several antennas from the literature operating in the 24 GHz ISM band. The comparison includes key metrics such as the number of radiating elements, realized peak gain, impedance bandwidth, center frequency, normalized size, substrate materials, and, most notably, aperture efficiency (eae-aea). In high-frequency applications such as automotive radar, maximizing the gain-to-size ratio is critical. Antennas must offer high directivity and low loss while remaining compact enough for integration into constrained vehicle body zones (e.g., bumpers, mirrors, grills). As such, aperture efficiency provides a more meaningful figure of merit than gain alone. It quantifies how effectively an antenna converts its physical area into radiated power and is defined by^42^ as:

11 $$\begin{aligned} e_{a}=\frac{G}{4\pi A_{phy} \lambda ^2} \end{aligned}$$

where G is the maximum gain of the antenna and $A_{phy}$ is the physical aperture (size) of the antenna. At the center frequency of 24.125 GHz, the proposed arrays demonstrate exceptionally high aperture efficiencies of 41.11% and 40.91% for Design I ($2 \times 5$ array) and Design II ($4 \times 5$ array), respectively.

**Table 3:** Comparison between the results of the proposed antenna with the other references.

Antenna structure	Radiating elements	Peak gain (dBi)	Imp. BW ($\%$)	$f_c$ (GHz)	Size ($\lambda _{0}\times \lambda _{0}$)	Dielectric substrate	Thickness (mm)	Aperture $e_{a}$($\%$)
Ref.^16^	$2\times 12$	14	5.49	23.600	$8.64\times 1.44$	RT/duroid 5880	1.578	16.07
Ref.^36^	$1\times 8$	11.1	16.9	23.700	$5.27\times 2.87$	Rogers RO4003C	3.26	6.78
Ref.^37^	$2\times 8$	16.4	37.7	24,000	$11.2\times 1.44$	Rogers RO3003	1.527	21.54
Ref.^38^	$3\times 3$	10.3	1.31	24.125	$1.58\times 1.44$	Rogers RO4350B	0.56	37.48
Ref.^39^	$4\times 4$	12.3	8.3	24,000	–	Arlon 25N	0.787	–
Ref.^40^	$2\times 8$	13.2	1.18	24.125	–	Rogers RO4350B	0.254	–
Ref.^41^	$1\times 18$	14.7	8.93	27.000	$7.83\times 1.28$	RT/duroid 5880	0.381	23.43
Design (I)	$2\times 5$	16	9.16	24.125	$5.07\times 1.52$	RT/duroid 5880	0.787	41.11
Design (II)	$4\times 5$	19.5	8.45	24.125	$5.27\times 3.29$	RT/duroid 5880	0.787	40.91

The high aperture efficiency achieved by the proposed arrays is mainly attributed to the antenna architecture rather than the optimization algorithm itself. The series-fed, proximity-coupled configuration ensures uniform amplitude and phase excitation across the radiating elements, thereby maximizing constructive interference in the main beam and improving effective aperture utilization. Moreover, the compact inter-element spacing and efficient aperture filling minimize inactive regions within the physical aperture. The proximity-coupled feed and low-loss power divider significantly reduce conduction and mismatch losses, leading to radiation efficiencies exceeding 95% and directly supporting the high aperture efficiency values. The role of the PSADEA algorithm is to efficiently identify optimal geometrical parameters that enable the antenna structure to operate close to its theoretical limit, rather than directly increasing the aperture efficiency.

These values outperform several state-of-the-art designs listed in the comparison. This performance is particularly impressive, given the relatively small number of radiating elements used: 10 elements for Design I and 20 for Design II, compared to larger arrays in some references that have lower efficiency. It is worth noting that the measured prototype sizes were increased by approximately 10% to accommodate edge-mounted 2.4 mm PCB connectors necessary for test integration. This additional area slightly reduces the calculated aperture efficiency. Nonetheless, even after accounting for this modification, both designs maintain superior performance relative to other compact arrays reported in the literature.

Design I achieves a realized gain of 16 dBi with only 10 elements, exceeding the 14 dBi gain reported in^12^, which uses 24 elements ($2\times 12$). Furthermore, the impedance bandwidth of 9.16% in Design I is significantly wider than the 5.49% bandwidth reported in^12^, offering better performance in dynamic environments and broader frequency coverage.

Design II achieves a realized gain of 19.5 dBi, which is 7.2 dB higher than the 12.3 dBi gain reported in^39^, despite using only two additional elements. This clearly illustrates the efficiency of the proposed feed network and optimization strategy.

In terms of aperture efficiency, Design I (41.11%) and Design II (40.91%) substantially outperform the other cited configurations. Only^27^ comes close in efficiency (37.48%), but it achieves a significantly lower gain (10.3 dBi) and a much narrower bandwidth (1.31%). These results have significant implications for 24 GHz automotive radar in IoT applications, where the antenna’s performance must be balanced with physical constraints and manufacturing costs. The high gain, broad bandwidth, and compact form factor of the proposed antennas make them particularly well-suited for applications such as:

- Short- and mid-range detection (Design I),
- Long-range forward sensing (Design II),
- Sensor fusion systems, where multiple antenna types are required in a limited physical space.

**Fig. 16:**
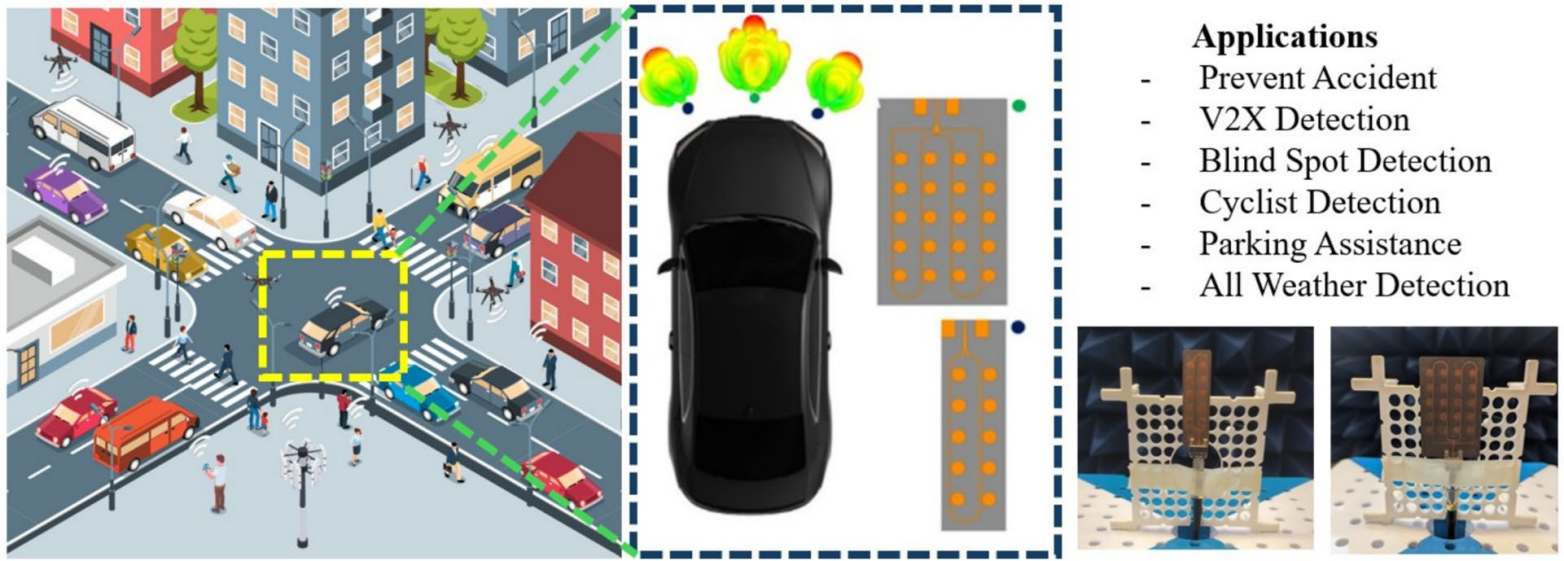
Overview of a proposed implementation of the automotive sensor to cloud-connected IoT devices (sensors, cameras, GPS trackers, telematics solutions) installed into vehicles to gather real-time data and provide a safer and smarter driving experience.

As demonstrated in Fig. 16, both designs are fabricated using Rogers RT/Duroid®5880, a material known for its low dielectric loss at millimeter-wave frequencies, ensuring consistent performance across environmental conditions. Combined with an AI-driven optimization method (PSADEA), the antenna designs offer a highly scalable, adaptable solution for next-generation radar sensors and IoT devices.

## Conclusion

This paper presented two optimized linear series-fed microstrip patch antenna arrays designed for 24 GHz automotive radar in IoT applications. The proposed 2 $\times$ 5 (Design II) and 4 $\times$ 5 (Design I) circular patch arrays employ proximity coupling and custom power divider networks to achieve efficient excitation, high gain, and compact form factors suitable for vehicle integration.

The antenna arrays were optimized using an AI-driven PSADEA algorithm, resulting in significant performance enhancements, including peak gains of 16 dBi (Design II) and 19.5 dBi (Design I), with aperture efficiencies exceeding 40% and measured radiation efficiencies above 95%. These designs also exhibit excellent impedance matching and stable radiation characteristics across the 23-25 GHz band.

While the proposed antennas are fixed-beam and do not support dynamic beam scanning, they remain well-suited for a wide range of short- to mid-range automotive radar applications, particularly those based on FMCW radar systems. Applications such as blind-spot detection, rear cross-traffic alert, and parking assist typically rely on fixed- or multi-beam architectures rather than full electronic scanning. In such scenarios, multiple fixed-beam modules (e.g., several Design II antennas placed side-by-side) or hybrid systems with analog beamforming can provide adequate angular coverage.

The fixed-beam architecture offers key advantages in these use cases: low cost, mechanical simplicity, high reliability, and ease of integration. While not intended for high-end beam-steering radar systems, the proposed antennas provide a practical and efficient solution for commercial automotive platforms where targeted, sector-specific coverage is sufficient.

Future work may explore integrating these arrays into multi-module radar systems, using switchable feed networks, or hybrid analog-digital beamforming to extend their functionality for scanning applications.

## Data Availability

The data that support the findings of this study are available from the corresponding author upon reasonable request.
